# Anaphylaxis Following a Mouse Bite in a Patient Sensitized to Mouse, Rat, and Hamster: A Case Report

**DOI:** 10.7759/cureus.101977

**Published:** 2026-01-21

**Authors:** Maako Kudo, Mayuko Goto-Umeki, Yutaka Hatano

**Affiliations:** 1 Department of Dermatology, Faculty of Medicine Oita University, Yufu, JPN

**Keywords:** anaphylaxis, hamster, mouse bite, rat, sensitization

## Abstract

We report a case of anaphylaxis in a man in his 30s following a mouse bite while working in an animal research facility. He developed generalized erythema, wheezing, and hypoxemia consistent with grade 2 anaphylaxis, which resolved after treatment with adrenaline, corticosteroids, antihistamines, and bronchodilators. Serum testing revealed specific IgE to mouse, rat, and hamster, despite no direct exposure to the latter two species. The patient had worked as an animal researcher for 15 years, exclusively handling mice. Before this episode, he reported mouse bites occurring every two to three years, which caused only localized itching. This case highlights the need for preventive measures in laboratory settings and for further investigation into sensitization routes and the potential cross-reactivity among rodent allergens.

## Introduction

Sensitization and allergy among laboratory workers exposed to rodents have been widely reported and remain an ongoing occupational health concern. In addition, the risk of rodent allergy in household settings has been increasing, partly due to the growing popularity of pets [1]. However, the routes and underlying mechanisms of sensitization to rodents in both laboratory workers exposed to rodents and individuals in household environments remain poorly understood.

To contribute to the discussion of these issues and potential countermeasures, we report a case of anaphylaxis, a severe, rapid-onset systemic allergic reaction that can be life-threatening and requires immediate treatment, caused by a mouse bite that occurred in an animal laboratory facility. In this case, serological testing revealed sensitization not only to the causative mouse but also to hamsters and rats, animals that the patient had never handled in experiments.

This case is noteworthy as it offers insights into possible sensitization pathways to laboratory animals and underscores the importance of developing effective preventive measures within animal research facilities.

## Case presentation

**Figure 1:**
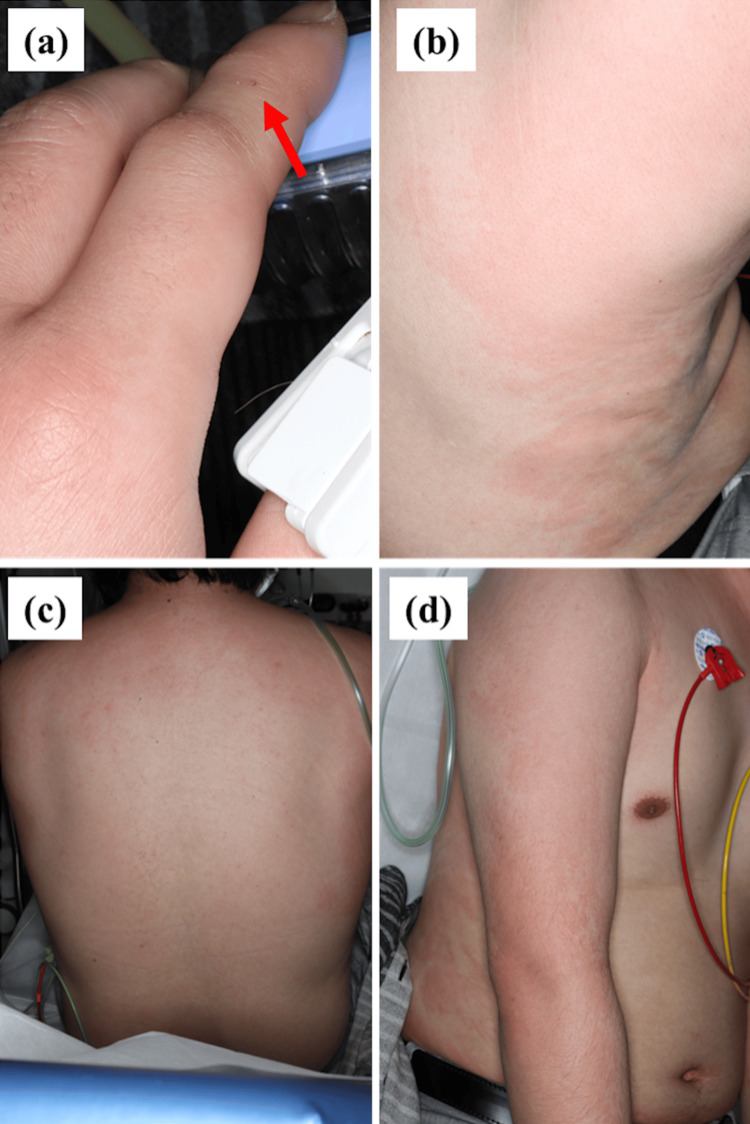
Clinical findings observed in the emergency room. (a) Left second finger. The red arrow indicates the site of the mouse bite. (b) Lateral chest. (c) Back. (d) Upper extremity.

A man in his 30s was bitten on the left second finger by a mouse during an animal experiment. Twenty minutes later, he developed redness all over his body and discomfort in his throat. Eighty minutes after being bitten, he presented to the emergency room of our hospital with swelling of the left second finger (Figure 1). On cutaneous examination, his face, neck, and extremities appeared flushed, and multiple map-like wheals were observed on the abdomen, back, and lateral chest (Figure 1). Wheezing was heard on auscultation. Heart rate was 100-110 beats/minute, blood pressure was 97/76 mmHg, and peripheral capillary oxygen saturation (SpO2) was 90%-93% in room air. Grade 2 anaphylaxis was diagnosed based on the skin and respiratory symptoms. In the emergency room, the patient was treated with a subcutaneous injection of 0.3 mg of adrenaline, an intravenous infusion of 125 mg of methylprednisolone succinate sodium, and D-chlorpheniramine maleate, as well as inhaled procaterol hydrochloride hydrate. Respiratory symptoms resolved completely within one hour, and the rash had nearly disappeared within 15 hours. The patient had a history of asthma and had been regularly using a combination inhaler containing budesonide and formoterol. There had been no asthma exacerbations in the preceding two years. He had a soy allergy but no other known allergies, including latex. After this episode, he handled mice only under anesthesia, and there were no further bites or recurrence of allergic symptoms. To our knowledge, no similar cases have been encountered in our facility.

A radioimmunosorbent test (RIST) and radioallergosorbent test (RAST) performed 7 days after being bitten showed a positive immunoglobulin (Ig)E-RIST value of 731 IU/mL (normal, ≤170 IU/mL) and positive IgE-RAST values of 0.51 IU/mL (class 1) for guinea pig epithelium, 1.88 IU/mL (class 2) for hamster epithelium, 13.7 IU/mL (class 3) for rat epithelium, and 92.5 IU/mL (class 5) for mouse epithelium. Although prick testing was not performed as the patient declined consent, these results suggested that the patient had become sensitized to rat and hamster as well as mouse.

The patient had worked as an animal experimenter for 15 years and had extensive experience handling mice but not rats or hamsters. Before this episode, he recalled being bitten by mice approximately once every two to three years on average. Although the bite did not cause swelling as seen in the present case, it did cause itching. The patient had never kept pets at home. He did not take any medications, including nonsteroidal anti-inflammatory drugs (NSAIDs), before or after the bite. He did not drink alcohol around the present allergic episode and has not engaged in any special exercise.

## Discussion

Although the timing and route of sensitization remain unclear, RIST and RAST results suggested sensitization not only to mice but also to hamsters and rats. Sensitization to mice was likely due to repeated mouse bites. In contrast, the patient had never handled rats or hamsters. Although inhalational sensitization to hamsters via exposure to hamster epithelium and hair has been reported [1], the air-conditioning systems for the mouse, rat, and hamster rooms in this facility were separate. Notably, all cages were cleaned in the same room, and the patient routinely participated in this study. Therefore, although difficult to prove, we suspect that sensitization to hamsters and rats occurred during cage-cleaning tasks, possibly through airborne exposure. Regarding cross-reactivity, the mouse antigen Mus m1 and the rat antigen Rat n1 are structurally similar and considered potentially cross-reactive [2], although whether such cross-reactivity contributed to the present case remains unknown. Cross-reactivity between mouse and hamster antigens has not been described, although simultaneous sensitization to both species has been reported [3,4].

To the best of our knowledge, the present case represents the third reported instance of sensitization to both mouse and hamster, although many cases may go unrecognized. In the first case, as in the present report, the patient was a laboratory researcher who appeared sensitized to hamster, rat, and mouse, but had a documented exposure history only for rats [3]. In the second case, the patient, also a laboratory researcher, showed sensitization to hamster and mouse but denied exposure to rats or to any animals other than mice at work [4]. The precise mechanisms underlying sensitization to multiple animals in the present case remain unclear. However, the possibility of sensitization to rodents in the absence of a clear contact history should be considered, particularly depending on the work environment.

Measures currently in place include wearing gloves to prevent allergen entry through contact or bites, proper removal of rodent excrement to minimize exposure to urinary allergens, and maintaining adequate laboratory ventilation and filter systems, along with mask use, to reduce exposure to airborne allergens [5,6]. The present case not only reaffirms the importance of these measures but also highlights the need to consider sensitization to other animals when an allergy to a specific animal is identified. At present, we are strictly implementing the preventive measures described in the case presentation; however, the precise extent of countermeasures required to be both necessary and sufficient remains unclear. The possibility of antigen cross-reactivity among mouse, rat, and hamster antigens should be addressed in future studies through the identification of sensitizing antigens. Finally, the most important limitation of this report is the absence of skin prick test results to confirm sensitization to hamster or rat.

## Conclusions

We report a case of anaphylaxis caused by a mouse bite in a patient who had been sensitized to multiple animals, including rats and hamsters, through unknown routes. Similar cases have involved researchers working in animal research facilities, underscoring the need for countermeasures such as improved environmental management in these settings. Further accumulation of evidence regarding the identification of sensitizing antigens and routes of sensitization is warranted.
